# Hearing people speak in different accents biases voice discrimination

**DOI:** 10.1038/s41598-025-13117-w

**Published:** 2025-08-21

**Authors:** Shane C. Santos, Aaliyah Kapadia, David R. Feinberg

**Affiliations:** Department of Psychology, Neuroscience and Behaviour, McMaster University, 1280 Main St West, Hamilton, ON L8S 4K1 Canada

**Keywords:** Psychology, Human behaviour

## Abstract

Voice discrimination is a fundamentally different task when matching utterances than when matching identity across different words. Discriminating between speakers of different languages makes the task even harder because unfamiliar languages contain different phonemes that are less easily matched. Discriminating between people with different accents may also be difficult as even if the same words are uttered, the phonemes are different. To test this, we created a set of voices using voice cloning that have the same or different identity or accent (UK, Poland, and China) and speaking different phrases. We tested how accent, sentences, and identify affected bias to conflate different identities as the same person. Contrasting identity between different and same increased bias to judge people as the same by about 62%. Contrasting accent between different and same independently increased bias to judge people as the same by about 10%. Contrasting between different and same sentences, changed bias to label people the same more when the accents were different than when they were the same. Our results are consistent with the idea that we are biased to think people typically speak with one accent. Thus, accents affect voice discrimination independently of language familiarity.

## Introduction

Individual recognition is a key factor in social behavior among many species. Humans use voices to recognize individuals^1^. Anatomical cues from the shape and size of the larynx, pharynx, and supralaryngeal vocal-tract (henceforth referred to as vocal-tract) provide a distinct acoustic signature for different people that is somewhat consistent across different types of utterances^2^. While large changes in acoustic features such as voice pitch and formant frequencies can shift vocal identity accuracy^1, ^familiarity with a speaker’s identity allows more flexible acoustic changes within an identity^3.^This, in-turn, increases accuracy in discrimination tasks. Voice recognition is not solely based on acoustic features, pitch and formant frequencies. Training in a particular language increases voice recognition accuracy^4^. The effects of training transfers between similar languages (e.g. English and German), but not across dissimilar languages (e.g. English and Mandarin), demonstrating that familiarity with the phonemes of a language gives leverage in voice identification independent of comprehension^5^. Furthermore, people with dyslexia have relatively more difficulty distinguishing speech from non-speech sounds^6^. Both adults and children with dyslexia exhibit lower voice-identification accuracy than do people without dyslexia^7–9^. Thus, language contributes to voice recognition at multiple levels.

In addition, early exposure to monolingual speakers versus bilingual speakers creates different sets of expectations about the statistical regularity of people speaking more than one language^10,11^. That expectation extends to accents^12,13^. Research using AI voice cloning supports the idea that we may be biased to falsely discriminate the same person as different people when that individual speaks in two accents^14,15^.

This bias can also be seen in infant studies, Mulak et al.^16^ found that infants are sensitive to linguistic and indexical changes in speech, particularly in vowel sounds. Our adult study is similar, but instead of using vowel height as an accent marker, we use full sentences for accented stimuli. To test whether expectations of accent congruency across utterances from a single speaker can affect voice discrimination, independent of familiarity with different English-language accents, we used AI-based voice cloning to separate the underlying identity (indexical cues) of the vocalizer from their speech patterns (linguistic cues). Next, we applied speech patterns from several people speaking English in three different accents (UK, Mandarin, and Polish) to the cloned voices. This yielded stimuli with the same or different identities, the same or different accents, and speaking the same or different phrases. In a 2 × 2 × 2 design (identity [same/different] x accent [same/different] x sentence [same/different]) we asked participants to listen to pairs of same-sex voices and decide whether they were the same person or a different person.

Following Mulak et al.^16^we predicted that one person speaking with different accents would be reported more often as two different people than one person speaking with the same accent. Conversely, we hypothesized that different people speaking with the same accent would more often be reported as the same person than two people speaking with different accents. We predicted these effects would extend to voice pairs speaking congruent and incongruent sentences as suggested by Wester & Karhila^14^.

## Methods and materials

All experimental protocols were approved by and carried out in accordance with McMaster University Research Ethics Board (MREB) protocol 6187. Informed consent was obtained from each participant prior to data collection. All data, analysis code, and the full outputs for all analyses for each study are publicly available on the Open Science Framework (https://osf.io/t25qa/).

### Stimuli

We used Retrieval-Based Voice Cloning (RVC)^17^ to apply accents to different identities. We isolated and extracted accented speech patterns from the recordings of people with UK, Mandarin, and Polish accents from the English language Speech Accent Archive^18^. Next, we superimposed the accented speech onto 4 men’s and 4 women’s voices from the CSTR VCTK Corpus^19^whose speech patterns were first stripped from their identities.

The result is a set of stimuli where several people speak in their own voice with speech patterns of two different people who speak with UK, Mandarin or Polish Accents. Eight different cloned identities (4 men and 4 women) spoke with a different person’s Mandarin accent, Polish accent, and British English accent. We produced 192 unique stimuli (4 target identities × 4 source identities × 2 sexes × 3 accents × 2 sentences).

### Participants

We used g-power^20,21^to calculate the number of participants required to find the smallest effect reported in Wester and Karhila^14^. Although this was an ANOVA, and we used a linear-mixed effects regression, given that our sample was so much larger than Wester and Karhila^14^we are confident in our ability to detect the hypothesized effects. The analysis suggested that only six raters for each stimuli pair would be needed to replicate their findings. To increase confidence in our ability to detect the effects we are looking for, we recruited 1000 participants to comfortably have 10 ratings for each of the 9710 stimuli pairs. Each participant identified a different random sample of 100 voice pairs, regardless of their country of origin. Participants were randomly recruited from people living in the UK, China, and Poland. We did not, however, balance our sample across those countries, as analyzing country of residence as a fixed-effects term was not an aim of the study. We also pre-screened out participants who self-reported hearing difficulties.

### Procedure

Mulak et al.^16^ show accent cues affected infant ability to identify voices, but only when the stimuli used only vowel sounds to remove speech patterns from identity. Thus, our study manipulated both identity and accent, in full sentences. The procedure yielded 9,710 combinations of voice pairs of all-within-sex combinations of identity, accent, and sentence, including comparing each sound to itself. Each participant evaluated a randomly selected subset of 100 pairs of a mix of male and female voices. The orders of stimuli pairs and voices within pairs were both randomized.

After providing informed consent, the software presented participants with a fixation cross and then they listened to the two voices in the pair. After the voices played, the participants were asked if they thought the two voices belonged to the same person or to two different people. The participants were required to press “s” if they thought the voices were the same person or “d” if they thought the voices were different people. After each trial, participants rated how confident they were in their answers on a 3-point scale (1 = Not at all confident; 2 = Somewhat confident; 3 = Very confident). After completing all the trials, participants rated their familiarity with each of the 3 accents (Mandarin/Polish/UK English), on the same 3-point scale (1 = Not at all familiar; 2 = Somewhat familiar; 3 = Very familiar). Finally, participants reconsented after being debriefed that the voices were cloned, synthesized, and not real. Participants were paid £9.75 per hour pro-rata for 15 min.

### Statistical methods

We conducted all analyses and plots using R and the following packages; dplyr 1.1.4, effects 4.2-2, emmeans 1.10.6, future 1.34.0, ggeffects 1.7.2, ggplot2 3.5.1, ggsci 3.2.0, gridExtra 2.3, jtools 2.3.0, kableExtra 1.4.0, knitr 1.49, lmerTest 3.1-3, robustHD 0.8.1, scales 1.3.0, sjPlot 2.8.16, tidyr 1.3.1, tidyverse 2.0.0, tools 4.4.2. All data, analysis code, and the full outputs for all analyses are publicly available on the Open Science Framework (https://osf.io/t25qa/). We used a binary logistic generalized linear mixed effects regression (GLMR) with a probit-link function. GLMER uses partial pooling to reduce the influence of unbalanced group sizes^22^.

Our response variable was whether (value = 1) or not (value = 0) participants responded that they thought the voices were same person. We effect-coded our binary predictors such that all *Different* conditions were − 0.5 (Different Identity, Different Accent, Different Sentence) and all *Same* conditions were 0.5 (e.g. Same identity, Same accent, Same sentence). We first summed familiarity across accents to reduce the number of random effects terms, and then z-scored the summed familiarity and confidence ratings.

We used a maximal random-effects structure^23,24^creating random slopes for all within-item and within-participant contrast. Our fixed effects were interactions between voice target identity (same/different), source accent (same/different), and sentence (same/different), as well controlling for confidence and accent familiarity. For our full code and output see supplementary materials (https://osf.io/t25qa/).

## Results

**Fig. 1:**
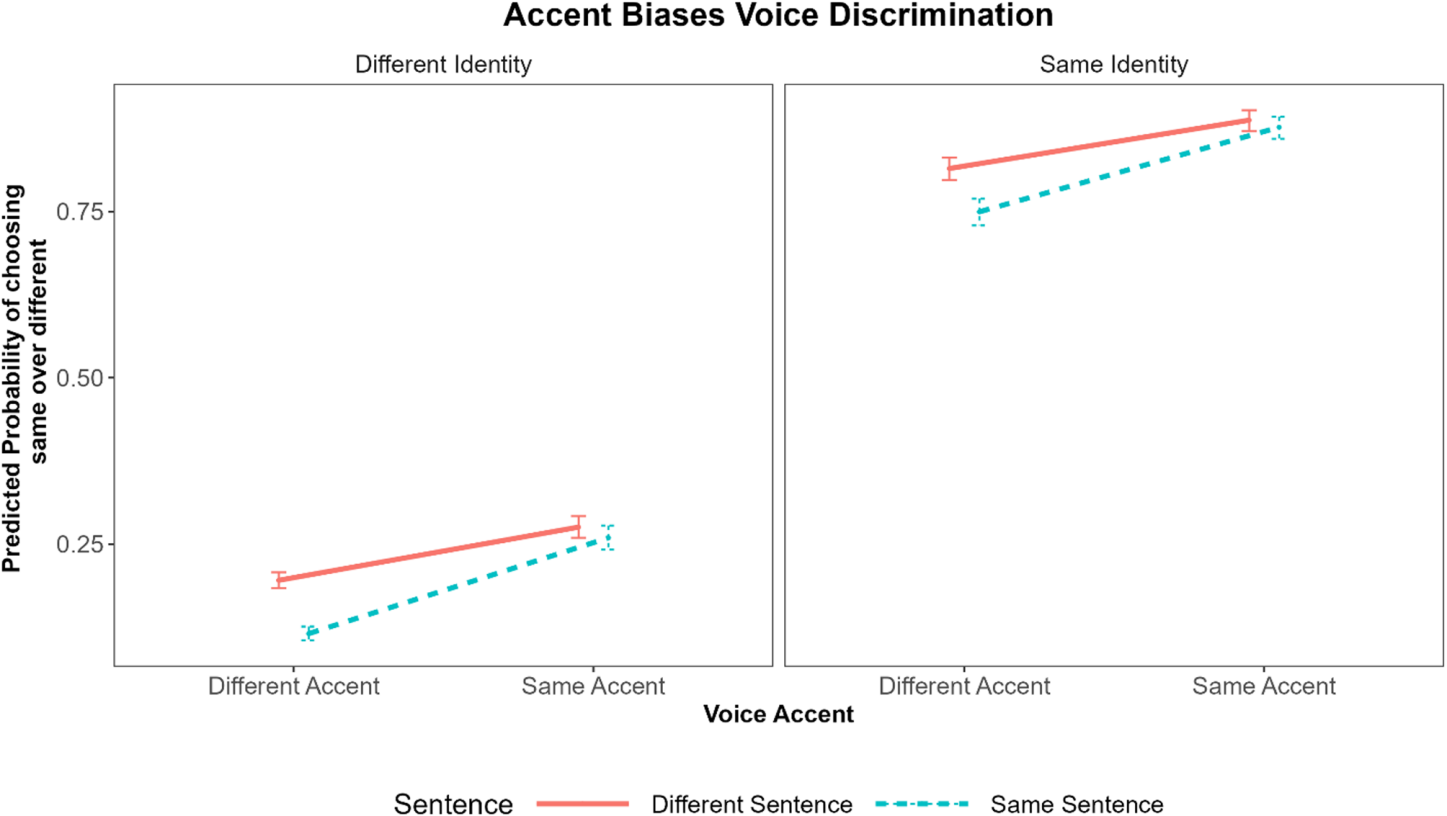
The estimated marginal probabilities of reporting voices as the same given hearing pairs of the same or different identity with the same or different accent speaking the same or different sentences. Same and different identities are in different panels. The x-axis indicating same and different accent. Solid red lines represent that different sentences were spoken. Dashed blue lines represent that the same sentence was spoken.

We found a three-way interaction between identity, accent, and sentence. When contrasting the difference between same and different identities, the bias to label someone as the same increased by about 62%. The main effect of identity was qualified by sentence, meaning that contrasting different- vs. same sentences reduced the bias to call people the same by about 4%. The interaction between identity and sentence, was qualified by accent, in that the reduction in bias to label people as the same granted by different sentences was offset when people used different accents by about 10%. All of the aforementioned effects and interactions were present when controlling for confidence and accent familiarity. Table 1 displays full details of the statistical output and Fig. 1 illustrates the interactions between identity, accent, and sentence.

**Table 1:** Table of fixed effects, detailing the coefficients, standard errors, and 95% confidence intervals z-values and for each effect and interaction.

	Estimate	Z value	2.5% CI	97.5% CI
Intercept	0.080	4.675	0.047	0.114
Identity	1.810*	61.499	1.752	1.868
Accent	0.405	22.168	0.369	0.441
Sentence	− 0.167	− 7.757	− 0.209	− 0.125
Confidence	− 0.196*	− 30.588	− 0.209	− 0.184
Familiarity	0.005*	0.291	− 0.026	0.035
Identity × accent	− 0.006	− 0.168	− 0.076	0.064
Identity × sentence	0.057*	1.591	0.013	0.127
Accent × sentence	0.231	6.524	0.161	0.300
Identity × accent × sentence	− 0.124	− 1.751	− 0.263	0.015

## Discussion

We tested if accent affects voice discrimination by measuring bias to conflate people with different identities, accents, and speech content. Our results suggest that accents affect voice discrimination by disrupting existing cognitive strategies for the task with uninformative biases.

Participants were highly accurate in discriminating people by their voices. The effects of identity on bias to conflate people were very large. There were independent main effects of accent on voice discrimination, suggesting that similar accents bias us to conflate different identities. In other words, we are biased to think that people with different accents are different people.

The bias to conflate different people as the same because they have the same accent was further qualified by people speaking same or different sentences. When people’s identities were the same, speaking the same or different sentences did not significantly change conflation bias depending on the accent. This may be because when people are using the same accent, we are better able to contrast phonemes from each other, rather than focusing on larger different across accent itself^4^.

However, when people’s accents were different, using *different* sentences increased conflation bias more than when they had the same accent. Using different sentences might reduce accent-driven conflation bias because the bias from differences between sentences are larger than biases differences between the same words in different accents. The difference in bias across same vs. different sentences when using same or different accents was slightly more pronounced among different identities than same identities. People were highly unlikely to conflate different identities speaking different accents if they spoke the same sentence.

If the results here were based only on language or accent familiarity, it would not matter who spoke the words, only that they were spoken in an unfamiliar accent. Furthermore, we controlled for familiarity with the accents by including z-scored ratings as a fixed factor. Thus, our findings are in addition to any effects of familiarity that might exist.

The effects of accent on voice discrimination represented on average, a 10% change in bias to conflate people as the same. These effects are independent of accent familiarity. Due to difference in sample sizes across countries, we did not analyze our data with respect to country of origin and recommend future research test whether more dissimilar accents have stronger effects than more similar accents.

Although sound quality and amplitude during testing could have influenced speech perception and therefore influenced the data, it is highly unlikely for that to have happened in a systematic fashion that would explain the interactions we reported. To address that, we accounted for the variance in responses due to sound quality and amplitude by using participant as a random effects term.

In summary, accents affect voice discrimination in addition to other factors such as familiarity. When discriminating voices humans need to be able to match low-level acoustic features of sounds, extrapolate and generalize that memory to new sounds that people speak. Our biases about people speaking different accents interfere with that process.

## Data Availability

All data, analysis code, and the full outputs for all analyses for each study are publicly available on the Open Science Framework (https://osf.io/t25qa/). Data are, however, available from the corresponding author upon reasonable request.
